# Wiktor Stein (1899–1979)

**DOI:** 10.1007/s00415-019-09467-x

**Published:** 2019-07-17

**Authors:** Jarosław Sak, Antoni Niedzielski

**Affiliations:** 1 Department of Ethics and Human Philosophy, Medical University of Lublin, ul. Staszica 4-6 (Collegium Maximum), 20-081 Lublin, Poland; 2 Psychology Unit of the Chair of Humanities, Medical University of Lublin, Lublin, Poland

**Fig. 1:**
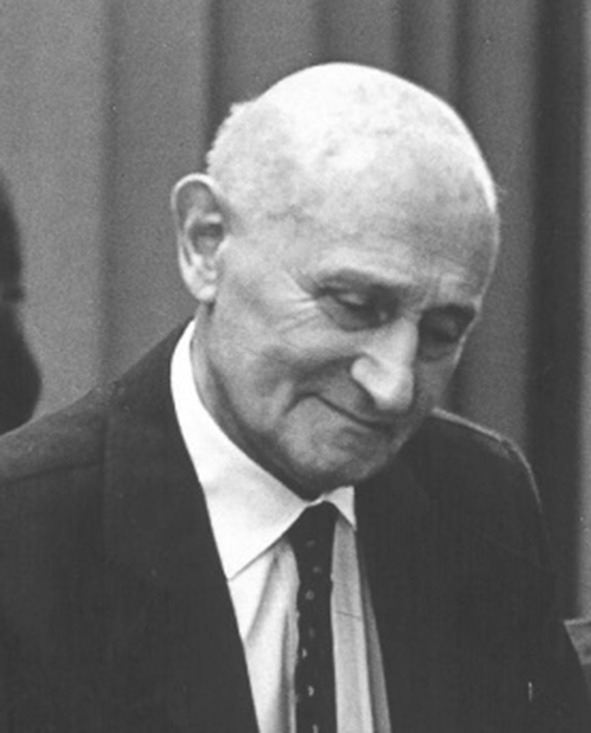
Wiktor Stein (1899–1979). The photo from Archives of the Medical University of Lublin

Wiktor Stein is considered one of the founders of Polish neurology after World War II (Fig. 1). He created the neurological clinic at the Mari Curie Skłodowska University (UMCS) of Lublin from scratch; using clinical experience gained at Lviv where Kazimierz Orzechowski (1878–1942) [1] also studied. In the scientific dimension, Stein was fascinated by the possibility of identifying centers involved in the process of regulating blood composition within the central nervous system. He was also the first to undertake research on the importance of leukocyte aggregation (leukergic) reactions in neurology. Those reactions were first discovered by Ludwik Fleck [2], who after World War II also worked at the same medical faculty in Lublin [3–5]. Currently, the phenomenon of leukocyte aggregation has some significance in explaining the etiology of neurological complications in the course of inflammatory diseases [6]. What is more, it has also become a subject of research for its connections with trauma and stress [7]. In the 1950s and 1960s, Stein also conducted research, of great clinical significance, on inflammatory changes in the cerebrospinal fluid in the course of tuberculous meningitis. To his successors at the neurology clinic in Lublin, Stein instilled the passion for searching for a drug that could cure multiple sclerosis (MS).

Wiktor Stein was born on November 28, 1899 in Lviv. His father, Maurycy Stein, was a doctor and a representative of the Jewish intelligentsia in Rohatyn (70 km from Lviv—south-eastern Poland before World War II), where during the second half of the nineteenth century the Jewish community comprised approximately 48% of the city’s total population [8]. In 1917, Stein passed his matura exam in Vienna and began his medical studies. On March 15, 1924, after graduating from the Faculty of Medicine at the University of Vienna, he obtained the title of Doctor of Medical Sciences.

After returning to Poland and the nostrification of his medical diploma, he was again promoted, at the Jagiellonian University in Krakow on February 4, 1926. The same year he started working at the Lviv State Hospital. In the period between January 1, 1927 and March 31, 1938, he worked at the Department of Nervous Diseases of the Lviv Hospital. In 1935 he completed an internship at the Neurological Clinic in Paris, headed by Georges Charles Guillain (1876–1961). Also in that year he became the head of the Lviv Polyclinic, where he worked until the outbreak of World War II.

In the years 1928–1936 he was the secretary of the Neurological and Psychiatric Society in Lviv, in the works of which he took an active part. During the Second World War he was in exile in Romania, where, as an outstanding violinist, he was a concertmaster in the Craiova Philharmonic Orchestra. After repatriation in 1946, he worked in Wroclaw as an assistant in the psychiatric clinic of the local University and as the head of the Neurological Department of the Provincial Hospital of Nervous Diseases. Two years later, he moved to Lublin, where he was entrusted with the management of the UMCS Neurological Clinic. He was nominated an associate professor in 1950 and a full professor in 1957. He held the position of head of the clinic until his retirement in 1970. In 1965 he became an honorary member of the French Neurological Society. His scientific achievements include over 40 works devoted to clinical matters. Wiktor Stein died on April 11, 1979.

Stein was conducting research on neurological diseases before World War II. Working at the neurological clinic in Lviv, together with Goldschlag, he published works on Swift’s disease (dermato-polyneuritis) [9] and after the War he conducted research in Lublin on humoral and vegetative regulation disorders occurring in the course of diseases of the nervous system. An important topic in his scientific portfolio was the leucocyte aggregation reaction, discovered in the 1940s by Ludwik Fleck. This reaction inspired researchers of subsequent decades to identify its clinical significance for various fields of medicine. Stein was the first scientist in the world to study its clinical applications in neurology [3, 4]. He was convinced that since the central nervous system regulates various vegetative functions (body heat, electrolyte economy), it should prove beneficial to look for the location of vegetative centers that could affect the morphological image of the blood. An essential element of this search was research carried out together with Fleck on the importance of central regulations behind leucocyte aggregation and leukocytosis and the mutual relationship of these two phenomena [3–5].

Publications from the 1950s and 1960s concerned research on changes in the cerebrospinal fluid in the course of tuberculous meningitis. Both pulmonary and non-pulmonary tuberculosis were huge epidemiological and clinical problems in post-war Poland. In his works, Stein used modern immunological methods; he studied the presence of specific antituberculin antibodies in the cerebrospinal fluid and considered the diagnostic value of these observations [10]. His own research on symptomatology in the diagnosis of tuberculous meningitis was published as a monograph.

Numerous original papers included those devoted to circulatory disorders in the spinal cord, carotid insufficiency, and rehabilitation of patients with sciatica. The Iron Curtain dividing Europe from the late 1940s made it difficult to exchange scientific ideas between East and West. However, archives of the Medical University of Lublin contain numerous traces of correspondence between Stein and scientists from the USA, France, Israel, Spain, Belgium, Netherlands and Germany asking him for his publications which were unavailable in full version in the West. Such requests were directed to Stein by, among others, Alfred J. Crowle of the Colorado Foundation for Research on Tuberculosis and Julio H. Garcia of the Medical College of Virginia. These archival documents are proof of a vivid exchange of scientific thought between Stein and many Western research centers.

Wiktor Stein was first and foremost a clinical neurologist, an excellent diagnostician, precise in clinical thinking. He was admired by colleagues and valued by patients. He combined the best traditions of Lviv neurological school, with a deep understanding of the need for rapid progress in neurology. The expression of the last was his constant and firm support for the development of neurosurgery. The ability of perspective vision and the correctness of assessing new phenomena was the result of the Professor's comprehensive, natural, humanistic and artistic mind.
